# Benchmarking of commercially available CHO cell culture media for antibody production

**DOI:** 10.1007/s00253-015-6514-4

**Published:** 2015-04-07

**Authors:** David Reinhart, Lukas Damjanovic, Christian Kaisermayer, Renate Kunert

**Affiliations:** Vienna Institute of BioTechnology, Department of Biotechnology, University of Natural Resources and Life Sciences,Vienna, Muthgasse 11, 1190 Vienna, Austria; BioMarin International Limited, Shanbally, Ringaskiddy County Cork, Ireland

**Keywords:** Chinese hamster ovary (CHO), Cell culture medium, Antibody production, Batch, Fed-batch, Medium benchmarking

## Abstract

In this study, eight commercially available, chemically defined Chinese hamster ovary (CHO) cell culture media from different vendors were evaluated in batch culture using an IgG-producing CHO DG44 cell line as a model. Medium adaptation revealed that the occurrence of even small aggregates might be a good indicator of cell growth performance in subsequent high cell density cultures. Batch experiments confirmed that the culture medium has a significant impact on bioprocess performance, but high amino acid concentrations alone were not sufficient to ensure superior cell growth and high antibody production. However, some key amino acids that were limiting in most media could be identified. Unbalanced glucose and amino acids led to high cell-specific lactate and ammonium production rates. In some media, persistently high glucose concentrations probably induced the suppression of respiration and oxidative phosphorylation, known as Crabtree effect, which resulted in high cell-specific glycolysis rates along with a continuous and high lactate production. In additional experiments, two of the eight basal media were supplemented with feeds from two different manufacturers in six combinations, in order to understand the combined impact of media and feeds on cell metabolism in a CHO fed-batch process. Cell growth, nutrient consumption and metabolite production rates, antibody production, and IgG quality were evaluated in detail. Concentrated feed supplements boosted cell concentrations almost threefold and antibody titers up to sevenfold. Depending on the fed-batch strategy, fourfold higher peak cell concentrations and eightfold increased IgG titers (up to 5.8 g/L) were achieved. The glycolytic flux was remarkably similar among the fed-batches; however, substantially different specific lactate production rates were observed in the different media and feed combinations. Further analysis revealed that in addition to the feed additives, the basal medium can make a considerable contribution to the ammonium metabolism of the cells. The glycosylation of the recombinant antibody was influenced by the selection of basal medium and feeds. Differences of up to 50 % in the monogalacto-fucosylated (G1F) and high mannose fraction of the IgG were observed.

## Introduction

Chinese hamster ovary (CHO) cells have become the standard mammalian host cell line for more than 70 % of recombinant protein pharmaceuticals on the market (Jayapal et al. 2007). This dominant position originates from their long history of use along with beneficial attributes such as their ability to express complex proteins, to conduct appropriate post-translational modifications, and their capability to secrete proteins into the cell culture supernatant, which facilitates downstream processing.

Since the first use of CHO cells for recombinant protein expression, production processes have steadily improved through advances in cell line development, clone screening and isolation, and optimization of basal media and feed supplements, as well as enhanced process monitoring and control. Compared to earlier processes, these developments allow 10-fold higher peak cell concentrations to be reached and product titers of up to 5 g/L are routinely achieved within 2 weeks of process duration (Jayapal et al. 2007; Yu et al. 2011). Optimized feed compositions and regimens improve culture longevity. They also lead to a more efficient cell metabolism, which reduces the formation of metabolites such as ammonium and lactate that may negatively affect growth, viability, product formation, and product quality (Butler 2005). Medium composition and development of an appropriate feed strategy is crucial, as excessive nutrient concentrations can be detrimental to cells due to component concentration dependent toxicity and high osmolality (Yu et al. 2011).

During the last years, several serum-free and chemically defined CHO-specific cell culture media have become available as well as some media systems that combine basal medium and feeds. However there is a need to understand the effects on cell metabolism and protein quality better when using different combinations of commercial basal media and feeds. In this study, we performed batch and fed-batch cultures with commercially available CHO cell culture media and feeds that were selected based on their novelty and widespread use. The lack of published data examining the way in which cell metabolism as well as antibody production and quality in a fed-batch culture are affected by different combinations of commercial basal media and feeds, encouraged us to test such combinations from two vendors. The influence on cell growth and antibody production of a recombinant CHO DG44 cell line was evaluated. Spent media were analyzed to evaluate the impact of the different media formulations on cell-specific nutrient utilization and formation of metabolic by-products. Finally, the effect of the different fed-batch strategies on the glycosylation of the recombinant IgG was also investigated.

## Materials and methods

### Maintenance culture and batch experiments

A CHO DG44 cell line (licensed from Cellca GmbH, Laupheim, Germany) was used as a model cell line. The cells were thawed and propagated in ActiCHO™ SM medium (GE Healthcare) supplemented with 6 mM L-glutamine and 30 nM methotrexate (both Sigma-Aldrich). In routine culture and batch experiments, cells were inoculated at a cell concentration of 2 × 10^5^ cells/mL, using a working volume of 35 mL in 125-mL Erlenmeyer shake flasks (Corning). Cultures were grown in an ISF1-X incubator shaker (Kuhner) at 37 °C, 140 rpm, 7 % CO_2_, and 90 % humidity. The cells were passaged every 3–4 days. Adaptation to new media was done through at least five consecutive passages, until a stable growth rate had been achieved. The media investigated were CD CHO, CD OptiCHO™, CD FortiCHO™ (all Life Technologies), Ex-Cell™ CD CHO (Sigma Aldrich), ProCHO™5 (Lonza), BalanCD™ CHO Growth A (Irvine Scientific), Cellvento™ CHO-100 (EMD Millipore), and ActiCHO™ P (GE Healthcare). After adaptation to the new media, batch cultures were started and sampled daily as described below. Cultures were terminated once the viability dropped below 60 %.

### Fed-batch cultures

Fed-batch cultures were grown in 500-mL Erlenmeyer shake flasks (Corning) at a starting volume of 100 mL. The inoculum concentration was 3 × 10^5^ cells/mL applying the same culture conditions as described for batch cultures. All fed-batch cultures were run in triplicates with feeding regimen as shown in Table 1. During the feed phase, cells adapted to ActiCHO P and OptiCHO media were supplemented with ActiCHO Feed A and Feed B (both GE Healthcare) and CHO CD EfficientFeed™ A (Life Technologies), respectively, according to the manufacturer’s recommendation. Additionally, the feeds were exchanged and used to supplement the other basal medium to investigate the effect on culture performance. Furthermore, FunctionMAX™ Titer Enhancer (Life Technologies) was added to fed-batch processes with both basal media to test its applicability as universal titer enhancer (Barrett et al. 2012). In all fed-batch experiments, a concentrated glucose solution (250 g/L; Sigma-Aldrich) was used to maintain the concentration of this nutrient above 3 g/L. Cultures were terminated once the viability dropped below 60 %.

**Table 1:** Feeding regimen for fed-batch cultures showing time of addition and amount of feed in percent of the culture volume

Basal medium	Start feed (day)	Feed 1 (day; %*w*/*v*)	Feed 2 (day; %*w/v*)
ActiCHO P	3	Feed A (daily; 3 %)	Feed B (daily; 0.3 %)
ActiCHO P	3	Feed A (daily; 3 %)	Feed B (daily; 0.3 %) and FunctionMAX (3, 5, 7; 3.3 %)
CD OptiCHO	3	EfficientFeed A (3, 5, 7, 9; 10 %)	None
CD OptiCHO	3	EfficientFeed A (3, 5, 7; 10 %)	None
CD OptiCHO	3	EfficientFeed A (3, 5, 7; 10 %)	FunctionMAX (3, 5, 7; 3.3 %)
CD OptiCHO	3	Feed A (daily; 3 %)	Feed B (daily; 0.3 %)

### Analyses

Each day, 2 mL of sample was drawn from the batch and fed-batch cultures for analysis of cell concentration, viability, antibody titer, and metabolite concentrations. The cell concentration was quantified using a Z2 Coulter Counter™ (Beckman Coulter), and the viability was determined by trypan blue dye exclusion with a Neubauer improved haemocytometer (MedPro).

Recombinant antibody concentration was determined by Bio-Layer Interferometry on an Octet™ QK (fortéBio). For IgG analysis, Protein A (ProA) biosensors (fortéBio) at 30 °C in phosphate buffered saline (PBS) with 1000 rpm agitation were used. Experimental curves were recorded for the individual samples, and data was processed and analyzed using the Octet data analysis software 6.4 (fortéBio). Finally, samples were quantified by alignment with a standard curve generated from serial dilutions of affinity-purified antibody product. The concentrations of key metabolites (i.e., glucose, glutamine, glutamic acid, lactate, and ammonium) were determined using a BioProfile™ 100 Plus (Nova Biomedical). Osmolality was measured in fed-batch cultures by freezing point depression using an Osmomat 030 (Gonotec).

Amino acid concentrations (except proline (Pro) and cysteine (Cys)) in media, feed supplements, and selected samples were analyzed at the end of the exponential growth phase and when a culture was terminated, using a high-performance liquid chromatography method. Briefly, protein in the samples was precipitated with 5-sulfosalicylic acid (Alfa Aesar) and the supernatant was subsequently cleared using a 0.2-μm filter unit (Sartorius). Free amino acids reacted with ortho-phthalaldehyde (Pierce) in an automated pre-column derivatization method and were then separated on a ZORBAX™ Eclipse Plus C18 column (Agilent) at 25 °C using a flow rate of 1.0 mL/min. After gradient elution using 50 mM sodium acetate pH 5.7 and acetonitrile (Merck), amino acids were excited at 340 nm and the fluorescence signal was detected at 450 nm. Samples were quantified applying 3-(2-thienyl)-DL-alanine (Fluka) as an internal standard.

Glycoprofiling was performed to investigate oligosaccharide distribution of antibody product upon harvest from fed-batch cultures. The cell suspension was centrifuged at 170 *g*, the supernatant clarified using a 0.22-μm filter (Express PLUS, Merck Millipore) and diluted with PBS to a concentration of 1 to 2 mg/mL. N-glycan profiles of the Fc regions of the monoclonal antibodies were determined by IdeS proteolytic digestion and electrospray mass spectrometry as described by Chevreux et al. (2011). Fc fragment signals with masses corresponding to N-glycan isoforms G0, G0F, G0F-GN, G1F-GN, G1F, G2F, G1F + SA1, G2F + SA1, G2F + SA2, Man5 to Man9, Man9 + Glc, and Man-9 + 2Glc were investigated, and their relative abundance rates were estimated from the intensity of the signals. Signals for N-glycan isoforms G1 and G2 were not amenable to quantification as their masses are only separated by 6 Da from the sodium adducts of G0F and G1F isoforms.

## Results

### Batch cultures

**Fig. 1:**
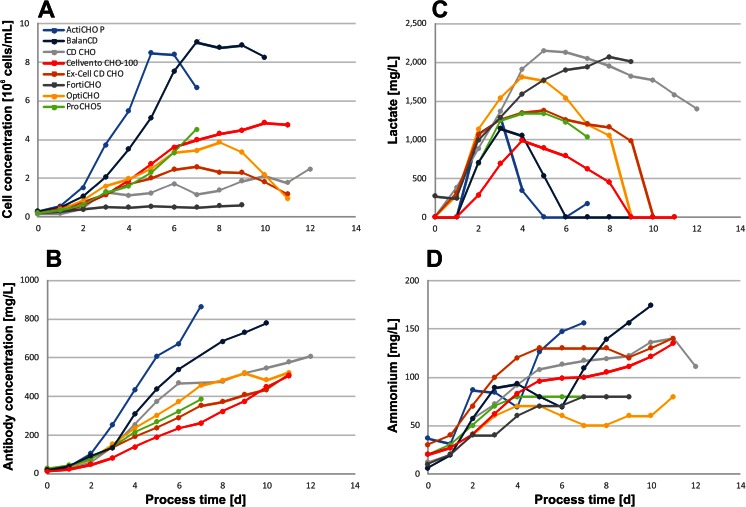
**a** Cell and **b** antibody concentrations as well as **c** lactate and **d** ammonium levels obtained with commercial CHO cell culture media in batch mode

In batch experiments, the impact of the culture medium on cell growth and antibody concentrations, as well as the process duration, was evaluated (Fig. 1a, b). A summary of the data generated from the batch cultures is given in Table 2. The highest cell concentrations were obtained in ActiCHO P (8.5 × 10^6^ cells/mL) and BalanCD (9.0 × 10^6^ cells/mL). In ActiCHO P, the cultures had a slightly higher growth rate and the maximal cell concentration peaked 2 days earlier than in BalanCD. However, in ActiCHO P, the cell concentration declined quickly after reaching its maximum, while a more pronounced plateau phase of about 3 days was observed in BalanCD. In ProCHO5, Cellvento CHO-100 and OptiCHO cell concentrations reached 4–5 × 10^6^ cells/mL. Although growth appeared to be similar in those three media until day 6, a drastic viability drop in ProCHO5 led to culture termination on day 7. In the other two media, the viability of the culture remained above 60 % during 4 further days. In Ex-Cell CD CHO, cells grew to cell concentrations (2.6 × 10^6^ cells/mL) which were about 70 % lower compared to ActiCHO P and BalanCD. The formation of small aggregates of 5 to 10 cells was observed in CD CHO as well as FortiCHO. In CD CHO, a maximum cell concentration of 2.5 × 10^6^ cells/mL was reached after 12 days. In FortiCHO, the cells barely grew and reached a maximum cell concentration of 6.0 × 10^5^ cells/mL during a process time of 9 days.

**Table 2:** Process relevant data from batch cultures

Medium	ActiCHO P	CD CHO	CD OptiCHO	Ex-Cell CD CHO	ProCHO5	BalanCD	Cellvento CHO-100	CD FortiCHO
Peak cell conc. (10^6^ cells/mL)	8.5	2.5	3.9	2.6	4.5	9.0	4.9	0.6
Antibody conc. (mg/L)	863	334	522	510	384	778	503	n.d.
μ exponential phase (1/d)	0.70	0.52	0.41	0.42	0.47	0.49	0.41	0.31
qP exp. phase (pg/(cell × day))	49.6	71.8	49.1	44.6	53.4	44.4	30.4	n.d.
qP whole process (pg/(cell × day))	41.9	42.7	35.2	36.8	49.9	29.3	28.5	n.d.
STY (mg/(L × d))	121	28	46	44	51	76	45	n.d.
qGlucose (pg/(cell × day))	−368	−482	−446	−434	−319	−149	−237	−647
qLactate (pg/(cell × day))	142	421	221	168	296	103	93	485
qGlutamate (pg/(cell × day))	−19	−6	−13	−2	8	22	−3	3
qGlutamine (pg/(cell × day))	−83	−85	−89	−80	−143	−72	−82	−99
qAmmonium (pg/(cell × day))	7	20	10	13	16	12	11	21

All values have been calculated as mean

*STY* space-time yield, *n.d.* not determined

Not surprisingly, antibody production was also influenced by the cell culture media (Fig. 1b). In general, high cell concentrations correlated well with high titers. Therefore, the highest IgG concentrations were reached in ActiCHO P with 863 and 778 mg/L in BalanCD. About 500 mg/L was achieved in OptiCHO, Ex-Cell CD CHO, and Cellvento CHO-100. Batch cultures in ProCHO5 and CD CHO reached 384 and 334 mg/L, respectively. Due to the low cell concentrations in FortiCHO medium, quantification of IgG was not applicable.

Lactate production differed considerably among the tested media. Typically, the maximum concentrations were reached during the initial 3 or 4 days of the cell culture as shown in Fig. 1c. The highest peak lactate concentrations were observed in CD CHO (2.2 g/L) and FortiCHO (2.1 g/L). In these media, the lactate concentrations remained high until the end of the culture. In OptiCHO, Ex-Cell CD CHO, BalanCD, Cellvento CHO-100, and ActiCHO P, lactate accumulated to concentrations between 1.0 and 1.8 g/L but was subsequently consumed. Ammonium levels increased continuously throughout the batch cultures, reaching concentrations of between 80 and 180 mg/L (Fig. 1d).

The high lactate concentrations of cultures grown in FortiCHO and CD CHO correlated well with elevated cell-specific lactate production rates of 485 and 421 pg/cell/day, respectively (Table 2). In addition, FortiCHO and CD CHO media triggered the highest specific glucose consumption rates. In all other media, lactate was consumed during the late stages of the culture, and 55–80 % lower specific lactate production rates, as well as lower glucose consumption rates, were observed compared with FortiCHO and CD CHO. The lowest glucose to lactate conversion rate, about 40 %, was seen in ActiCHO P, Ex-Cell CD CHO, and Cellvento CHO-100. At the other extreme, cells in ProCHO 5 converted 92 % of the consumed glucose to lactate. The mean specific ammonium production rates in the different media were 14 ± 7 pg/cell/day. The specific rates were highest for cultures in CD CHO and FortiCHO, media where no high cell concentrations were reached.

Residual amino acid concentrations were quantified at two time points: at the end of the exponential growth phase and at culture termination when the viability was below 60 %. Concentrations were compared to the composition of the cultivation medium used. To identify limiting amino acids, an arbitrary cutoff of 20 % compared with the initial medium concentration was set. As shown in Table 3B, glutamine (Gln) was exhausted in all media at the end of exponential growth phase. Limitations of other amino acids varied among the tested media. Tyrosine (Tyr), serine (Ser), aspartic acid (Asp), and asparagine (Asn) appeared most critical. Increased concentrations of alanine (Ala) and glycine (Gly) were observed in all media. Glutamic acid (Glu) concentrations increased only in ProCHO5, Ex-Cell CD CHO, and BalanCD while Asp concentrations increased in Ex-Cell CD CHO and Cellvento CHO-100.

**Table 3:**
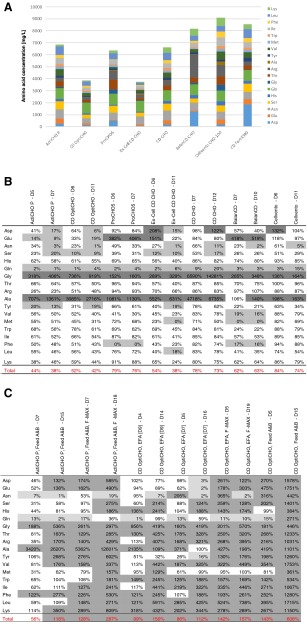
(A) Cumulative amino acid concentrations without proline and cysteine of commercial CHO cell media. Percentage of amino acids in the sample compared to the initial medium concentration at the end of the exponential growth phase and at culture termination of (B) batch and (C) fed-batch experiments. Dark gray scale indicates amino acids which increased during the cultivation (cutoff = 120 %); light gray scale highlights amino acids which corresponded to ≤20 % of the initial medium concentration

### Fed-batch cultures

During the fed-batch experiments, we aimed to investigate the influence of feed additives on the culture performance using two basal media based on their performance in the initial batch culture. Moreover, we evaluated whether feed supplements had similar effects when used with different basal media. The different media and feed combinations are shown in Table 1. During batch cultivation, the highest cell and antibody concentrations were obtained in ActiCHO P and BalanCD medium. However, supplementary fed-batch experiments using BalanCD and the medium’s recommended feed substrate, Feed 1 (Irvine Scientific), resulted in lower cell concentrations compared to the batch culture despite a 5-day prolonged process (data not shown). Compared with the batch culture, the fed-batch increased the IgG yield by about 40 % to 1.2 g/L. In contrast, OptiCHO performed well with more than one feed option (Barrett et al. 2012). These preliminary experiments convinced us to apply OptiCHO for subsequent fed-batch experiments including various feed combinations.

**Fig. 2:**
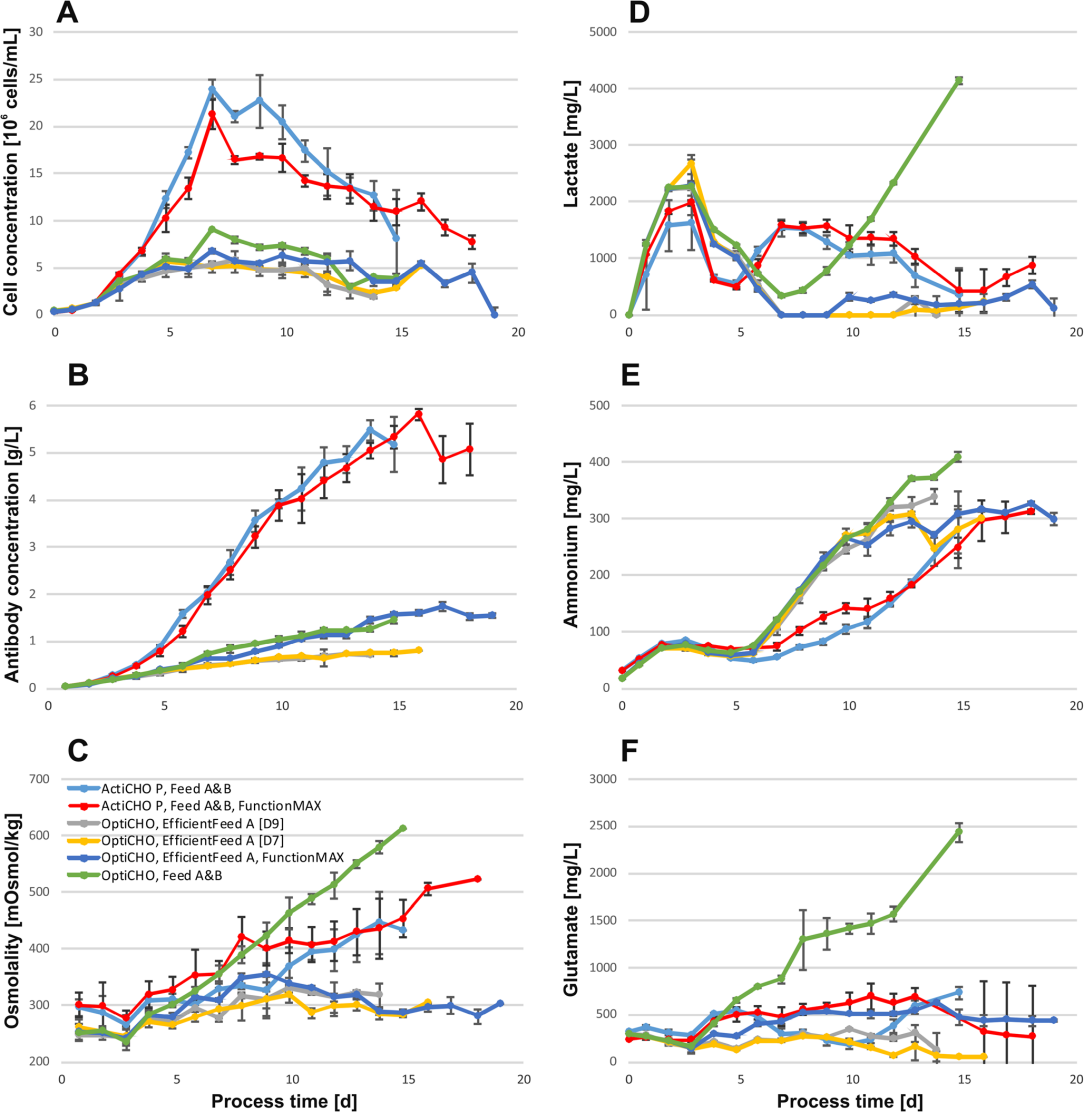
**a** Cell and **b** antibody concentrations, **c** osmolality, **d** lactate, **e** ammonium, and **f** glutamic acid concentrations obtained with commercial CHO media in fed-batch mode. All values represent the mean of three replicate experiments. *Error bars* show 1 standard deviation

The composition of basal media and feeds had a substantial effect on peak cell concentrations and antibody titers as shown in Fig. 2a, b. The highest cell and product concentrations of 2.39 × 10^7^ cells/mL and 5.5 g/L were achieved in cultures grown in ActiCHO P supplemented with Feed A and B. Feeding these cultures additionally with FunctionMAX altered the process only marginally reaching peak cell concentrations of 2.13 × 10^7^ cells/mL and IgG titers of 5.8 g/L. Fed-batch cultures in OptiCHO achieved 40 % or less of the peak cell concentration observed in fed-batch cultures with ActiCHO P as a base medium. Cell growth was lowest when cultures were grown in OptiCHO and fed with EfficientFeed A only, irrespective of whether the feed was added during 7 or 9 days (5.76 or 5.74 × 10^6^ cells/mL, respectively). When cultures in OptiCHO were supplemented with Feed A and B, 25 % higher cell concentrations (9.03 × 10^6^ cells/mL) were obtained than when supplemented with EfficientFeed A and FunctionMAX together (6.80 × 10^6^ cells/mL). In OptiCHO, the final titers were highest when combining EfficientFeed A and FunctionMAX as feed substrates (1.7 g/L). Slightly less product (1.5 g/L) was obtained when cells were supplemented with Feeds A and B, however, with the benefit of a 4-day shorter culture duration. Substantially lower product concentrations where observed in OptiCHO cultures fed with EfficientFeed A alone, irrespective of whether feeding lasted until day 7 or 9 (0.8 and 0.7 g/L, respectively).

All fed-batch cultures reached their plateau phase after a process time of 5 to 7 days and the specific growth rate declined thereafter (data not shown). During the initial 7 days, the average growth rate was highest in ActiCHO P fed-batch cultures supplemented with Feed A and B (0.63 ± 0.00 day^−1^), and ActiCHO P fed with Feed A and B together with FunctionMAX (0.65 ± 0.02 day^−1^) as shown in Table 4. In OptiCHO fed-batch cultures, the average growth rates were about 30 % lower. When supplemented with Feed A and B, cells in this medium grew at an average rate of 0.46 ± 0.01 day^−1^; this was followed by cultures fed with EfficientFeed A and FunctionMAX (0.43 ± 0.03 day^−1^). Finally, cultures supplemented with EfficientFeed A had growth rates of 0.38 ± 0.02 and 0.40 ± 0.04 day^−1^ when fed during 7 and 9 days, respectively.

**Table 4:** Process relevant data from fed-batch cultures

Medium	ActiCHO P, Feed A&B	ActiCHO P, Feed A&B, F-MAX	CD OptiCHO, Eff. Feed A (D9)	CD OptiCHO, Eff. Feed A (D7)	CD OptiCHO, Eff. Feed A, F-MAX	CD OptiCHO, Feed A&B
Peak cell conc. (10^7^ cells/mL)	2.39	2.13	0.58	0.57	0.68	0.90
Antibody conc. (g/L)	5.48	5.82	0.72	0.80	1.74	1.46
μ exponential phase (1/d)	0.63	0.65	0.40	0.38	0.43	0.46
qP exp. phase (pg/(cell × day))	51.2	51.2	31.7	29.9	36.7	39.3
qP whole process (pg/(cell × day))	43.9	46.9	27.4	28.8	35.6	31.0
STY (mg/(L × d))	344.7	281.8	51.4	50.0	81.5	97.3
qGlucose^a^ (pg/(cell × day))	−312.2	−303.0	−252.6	−259.5	−312.9	−294.1
qLactate^a^ (pg/(cell × day))	59.2	52.3	56.5	55.1	33.0	134.3
qGlutamate^a^ (pg/(cell × day))	−10.9	−14.0	−28.4	−21.0	−12.6	−8.0
qGlutamine^a^ (pg/(cell × day))	−30.4	−22.7	−30.9	−31.4	−32.3	−30.3
qAmmonium^a^ (pg/(cell × day))	3.2	3.2	8.2	5.4	4.7	7.6

*STY* space-time yield

^a^Mean values calculated starting from feed start (day 3) over the duration of the complete fed batch culture

The highest cell-specific antibody productivities were obtained in ActiCHO P fed-batches. Processes using Feeds A and B yielded an average qP of 51.2 pg/cell/day during the initial 7 days (Table 4). A further supplementation with FunctionMAX did not increase the average qP (51.2 pg/cell/day). Fed-batch cultures in OptiCHO reached 20 to 40 % lower specific productivities compared with the ActiCHO P cultures. Supplementation with EfficientFeed A and FunctionMAX or with Feeds A and B resulted in similar values of 36.7 and 39.3 pg/cell/day, respectively. The lowest specific productivities were observed when OptiCHO was only supplemented with EfficientFeed A for 7 (29.9 pg/cell/day) or 9 days (31.7 pg/cell/day). The volumetric productivity (space-time yield, STY) differed among the fed-batch cultures as shown in Table 4. This is a consequence of the different cell concentrations and cell-specific productivities. The highest STY was obtained in ActiCHO P fed with Feed A and B (335 mg/L/day) or further supplemented with FunctionMAX (282 mg/L/day). The second best performing strategies, but already 70 % lower, were OptiCHO supplemented with Feed A and B alone (97 mg/L/day) or combining EfficientFeed A with FunctionMAX (82 mg/L/day). Another 50 % reduced STY was obtained when OptiCHO was fed with EfficientFeed A for 7 or 9 days (50 and 51 mg/L/day, respectively).

The concentrations of lactate, glutamic acid, and ammonium are shown in Fig. 2d–f. Lactate peaked on day 2 or 3 in all cultures and was later consumed to levels below 1 g/L. After day 6, lactate concentrations were considerably lower in OptiCHO fed-batches than in ActiCHO P. However, when cells in OptiCHO were fed with Feed A and B, a continuous increase of lactate to 4.2 g/L was observed after the end of exponential growth phase. Additionally, using this combination, up to 2.4 g/L glutamic acid accumulated, whereas in all other fed-batch cultures, its concentration remained below 0.7 g/L. Glutamine was readily consumed within the first 3 to 5 days in all fed-batch cultures (data not shown). Ammonium accumulated following a similar trend in all fed-batch cultures during the initial 5 days, after which the concentration of this metabolite increased faster in OptiCHO cultures than in ActiCHO P. This was caused by the elevated cell-specific ammonium production in OptiCHO cultures during the plateau phase. From day 7 until day 10, it was up to six times higher in OptiCHO cultures than in ActiCHO P (data not shown). This resulted in similar absolute ammonium concentrations of 0.30 to 0.34 g/L in both media despite the lower cell concentrations in OptiCHO. Notably, cultures grown in OptiCHO and supplemented with Feed A and B reached the highest ammonium concentrations (0.41 g/L).

The evaluated feed combinations resulted in substantial osmolality differences as shown in Fig. 2c. The highest values were observed in OptiCHO fed with Feed A and B. In this case, the osmolality rose above 400 mOsm/kg on day 9; during the following 6 days, it increased further, reaching a maximum of 610 mOsm/kg, whereas the osmolality in all other fed-batch cultures in OptiCHO remained 40 to 55 % lower at levels of 280 to 350 mOsm/kg. The ActiCHO P fed-batch cultures fed with Feed A and B or additionally with FunctionMAX also passed a threshold of 400 mOsm/kg on days 13 and 8, respectively. However, in case of feeding with Feed A and B only, a maximum of 440 mOsm/kg was reached on day 14; when FunctionMAX was added, the osmolality peaked on the last day (day 18) at 520 mOsm/kg.

The residual percentage of amino acids compared to their original concentration in the respective cell culture medium at the end of the exponential growth phase and at the end of the process is shown in Table 3C. The residual concentrations in all fed-batch cultures barely dropped below critical levels. Gln was the only amino acid that decreased below 20 % of its original medium concentration in all fed-batch experiments at the end of the exponential growth phase. Additionally, in OptiCHO cultures fed with EfficientFeed A, low levels of Asp, Glu, and Tyr were observed. During the feed phase, several amino acids accumulated, especially toward the end of the process. The medium/feed combination with the lowest amino acid accumulations was ActiCHO P with Feeds A and B where 3 (exponential phase) and 10 (harvest of culture) amino acids exceeded their original medium concentrations. This was followed by OptiCHO fed with EfficientFeed A during 9 and 7 days, where 8/13 and 10/11 amino acids, respectively, accumulated. When cultures in ActiCHO P were fed with Feed A and B together with FunctionMAX, 12/16 amino acids accumulated. When the cell line was grown in OptiCHO and fed with EfficientFeed A as well as FunctionMAX, 15/16 amino acids accumulated. The highest residual amino acid concentrations were observed in fed-batch cultures in OptiCHO medium supplemented with Feed A and B, where 15/18 amino acids were overfed.

**Fig. 3:**
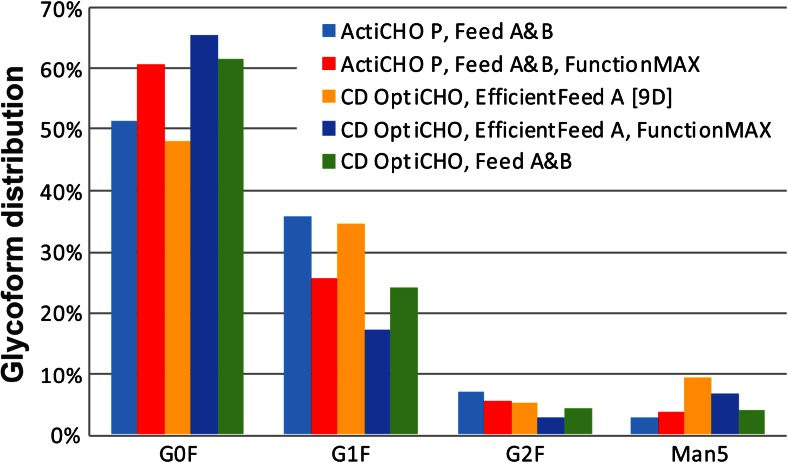
Antibody glycoform distribution at harvest during fed-batch cultivation in different commercial CHO media

The impact of the diverse fed-batch combinations on product glycosylation was investigated upon culture harvest. The glycan pattern of the antibody was determined at high resolution, and all major N-glycans were detected with good signal-to-noise ratios (data not shown). As illustrated in Fig. 3, antibody glycosylation varied among the fed-batches. However, the main glycan structures had a similar distribution with core fucosylated agalacto glycans (G0F) being most prevalent (48–66 %), followed by monogalacto (G1F; 17–36 %) and digalacto (G2F; 3–7 %) glycans. The percentage of high mannose structures (Man5) ranged between 3 and 9 %.

## Discussion

During the intial study in batch culture, a CHO DG44 cell line expressing a recombinant IgG was grown in eight commercially available CHO cell culture media. Our main goal was to investigate the effect of different CHO cell culture media on cell metabolism and antibody production. During adaptation to the different media, aggregates of 5 to 10 cells were observed in CD CHO and FortiCHO. Despite showing constant growth rates in the adaptation cultures, the cells in these two media did not grow to high concentrations. Under suboptimal culture conditions, cells may undergo apoptosis, which causes the release of DNA from dead and decaying cells. This promotes cell-cell adhesion and ultimately leads to the formation of cell clusters or aggregates. Cell aggregation is detrimental to a bioprocess and has been shown to reduce the specific growth rate by up to 50 % and increase cell-specific death rates due to exerting higher shear forces on large aggregates than on single cells (Renner et al. 1993). In our case, cell aggregation was less pronounced during the adaptation phase, most likely due to the regular medium exchange two times per week and relatively high viabilities of >95 %. Since viability and cell growth seemed reasonable during the adaptation phase, the occurrence of even small aggregates might be a good indicator of cell growth performance in subsequent high cell density cultures where the negative effect of cell clustering may be enhanced.

Cell and antibody concentrations during batch experiments correlated well (Fig. 1a, b). Consequently, high cell concentrations resulted in the highest titers. Lactate production differed considerably among the cultures. Media with high cell-specific lactate production rates coincided well with high absolute concentrations (Fig. 1c and Table 2). The increasing lactate concentration, perhaps in combination with the formation of small aggregates during medium adaptation in CD CHO and FortiCHO, might also provide evidence for suboptimal cell performance in these media. Lactate is a major by-product of cell culture that causes acidification of the medium. In bioprocesses, the pH is neutralized by the addition of base which increases the osmolality and can lead to a reduction in cell growth, viability, and recombinant protein yields. One way to reduce lactate formation is to maintain a low residual glucose concentration, which has been shown to shift the cell metabolism to more efficient pathways in BHK (Cruz et al. 1999), hybridoma (Zhou et al. 1995), and CHO (Fan et al. 2014) cell cultures. For BHK cells, Cruz et al. (1999) showed that this shift occurs at glucose levels below 1 mM. Yet all selected media contained glucose above this level, which may account for the high glycolytic activities together with high initial lactate productivities that we associated with an increased growth of CHO cells, similar to previous observations (Li et al. 2012).

The energetically inefficient metabolism which was observed may be attributed to the Crabtree effect. Here, elevated glucose concentrations inhibit oxygen consumption, which results in aerobic glycolysis and lactate formation. It has been shown that some cancer cells can regulate their energy metabolism and reversibly switch between fermentation and oxidative metabolism, depending on the absence or presence of glucose and environmental conditions (Diaz-Ruiz et al. 2011). A pronounced cell-specific glycolysis rate was determined, especially for batch cultures in FortiCHO and CD CHO (Table 2), at which the main fraction of glucose supposedly did not enter the citric acid cycle and oxidative phosphorylation but was merely metabolized via pyruvate to lactate (Table 2). A Crabtree effect seemed reasonable since higher glucose concentrations coincided with higher lactate production rates (data not shown). These rates decreased with decreasing glucose concentrations until they became negative, indicating lactate consumption, when glucose was used up. Glucose concentrations remained high in CD CHO and FortiCHO and, therefore, a persistent glucose-induced suppression of respiration and oxidative phosphorylation (Crabtree effect) resulted in continuous production of lactate. Another possible explanation for this could be an unbalanced nutrient supply for the respective cell line, which might lead to cellular stress and suboptimal growth which in turn leads to increased secretion of by-products. Indeed, an unbalanced supply of glucose and amino acids has recently been shown to lead to high lactate and ammonium concentrations, which inhibited CHO cell growth in a fed-batch culture (Fan et al. 2014). Interestingly, cells in CD CHO and FortiCHO also had the highest specific ammonium production rates among all investigated cultures (Table 2). Concerning amino acids, we observed that 38–76 % were still abundant at culture termination compared to the total initial medium concentration (Table 3B). Higher amino acid consumption rates often coincided with higher final antibody titers and higher viable cumulative cell days (data not shown). Therefore, not only the concentration of single amino acids but also the reduction of the total amino acid supply may serve as an indicator for their balance. Another potential explanation, although not analyzed in this study, could be related to copper, which has been identified as a critical medium component in CHO cell cultures. Too low concentrations of copper in culture media can lead to reduced cell concentrations and the accumulation of lactate, with no subsequent consumption of this metabolite. On the contrary, high copper concentrations may lower the cell-specific glucose consumption rate and result in less lactate production (Luo et al. 2012).

Quantification of residual amino acid concentrations in the media is important as their availability is seen to be strongly linked to recombinant protein expression (Jordan et al. 2013). Remarkably, at the end of the exponential growth phase, cells had consumed roughly half and in some media only less than a quarter of the total initial amino acids (Table 3B). Despite some variations, Gln was consumed in all media and occasionally Glu, Tyr, Ser, Phe, Val, and Met were also consumed. As Tyr and Ser were proposed to be important factors for cell growth (Kim et al. 1998; González-Leal et al. 2011; Jordan et al. 2013), and Met has been described as an important donor of sulfur and methyl groups for efficient recombinant protein production (Kim et al. 2005), it would be interesting to see whether elevating their concentrations prolongs the exponential growth phase. Between the end of the exponential growth phase and the end of the process, the total amino acid concentration dropped by another 3 to 16 %, and several of the 18 amino acids which were analyzed reached limiting concentrations. Although amino acid utilization is specific for individual cell lines, culture conditions, and biological products (Jayme 1991), we and others repeatedly found Asn (in six media), Ser (four), Tyr (three), and Asp (three) to be limiting (Chen et al. 2000; Altamirano et al. 2006; González-Leal et al. 2011; Jordan et al. 2013). Therefore, somewhat higher concentrations might be beneficial in terms of cell growth and productivity. As observed previously (Chen et al. 2000; Xing et al. 2011), Ala and Gly were produced in all media. Gly has been shown to improve CHO cell growth (Chen and Harcum 2005), but high concentrations of Ala were found to affect biomass production negatively by signaling that intermediates produced from TCA cycle are abundant. Thus, only a minimal supply of Ala should be applied to prevent inhibition of pyruvate kinase and the TCA pathway (Xing et al. 2011).

In summary, our results show that high amino acid concentrations alone are not sufficient to support high cell growth and antibody production. This was further corroborated by testing a chemically defined in-house prototype medium in which up to three-fold higher amino acid concentrations were used. Cell culture in this prototype medium did not result in a corresponding improvement of biomass or IgG concentration (data not shown). Likewise, ProCHO5, CD CHO, and ActiCHO P contained similar total amino acid concentrations, but the outcome of the respective cell cultures differed significantly with respect to cell concentration and IgG titer. Despite the fact that the total amino acid consumption was found to be surprisingly low in some media (Table 3A), which certainly still allows for optimization, our data suggest that it is not the total amount of amino acids that is critical for cell growth and recombinant antibody production but rather a balanced medium composition that meets the needs of the cell line used.

In a follow-up study, the impact of the basal medium formulation on fed-batch cultures and the effect of different feed supplements on cell growth, metabolism, recombinant protein production, and IgG glycosylation were evaluated. Fed-batch experiments demonstrated the beneficial effect of feed solutions tailored to certain commercial media. Compared with the batch cultures, the peak cell concentrations increased by 280 and 230 %, titers by 670 and 330 %, and volumetric productivities (STY) by 260 and 200 %, and finally, the cell culture process was extended by 11 or 8 days in ActiCHO P and OptiCHO media, respectively. In general, nutrient supplementation of the basal media had a positive effect on cell growth and titers, although a well-balanced basal medium was fundamental, even for the best performing feed supplements, to reach peak cell concentrations around day 7 and maintain antibody secretion until the end of the cell culture (Fig. 2).

ActiCHO P supplemented with Feed A and B yielded higher titers than OptiCHO in combination with the same feeds. OptiCHO with the addition of the recommended feed (EfficientFeed A) did not increase titers or cell concentrations in the same manner as ActiCHO P with Feed A and B. Instead, the combination of OptiCHO and EfficientFeed A resulted in the least productive fed-batch with a volumetric productivity of 50 mg/L/day (Table 4). In contrast, propagation in ActiCHO P combined with Feed A and B or an additional supplementation with FunctionMAX reached almost sevenfold higher values. Other combinations of OptiCHO such as feeding with EfficientFeed A and FunctionMAX or Feed A and B elevated the STY about twofold indicating that the underlying basal medium is important for the success of the applied feed strategy. As shown by the results from OptiCHO, a feeding strategy, for example, with the use of either FunctionMAX or Feed A and B can still boost the volumetric productivity. Yet, an optimized basal medium and feed combination resulted in an up to sevenfold increase, as shown by the example of ActiCHO P with the tested feed combinations.

Proliferating cells often ingest more nutrients than are bioenergetically needed and shunt metabolites into biosynthetic pathways (Bauer et al. 2004; Shaw 2006; DeBerardinis et al. 2008; Vander Heiden et al. 2009). The high glycolytic flux triggers the cells to the excessive accumulation of pyruvate, which is secreted as lactate (DeBerardinis et al. 2008). We found remarkably similar glucose consumption rates (Table 4), but substantially different cell-specific lactate production rates in the investigated media and feed combinations. The feeds had a drastic influence causing a fourfold difference between the lowest lactate production rate in OptiCHO supplemented with EfficientFeed A and FunctionMAX compared to the highest production rate when cultures in OptiCHO were fed with Feed A and B. A possible explanation is that in this basal medium, cells did not grow sufficiently fast to consume all the nutrients supplied by the daily addition of Feed A and B. Nutrient overfeeding may shift cells to a less efficient energy metabolism and led to high osmolality, which can trigger higher lactate productivities as discussed further in this section.

The specific consumption rate of glutamine, which has been described to contribute substantially to lactate production (Dean and Reddy 2013), was similar in all media. The development of ammonium concentrations showed substantial differences in fed-batch cultures grown in either OptiCHO fed or ActiCHO P (Fig. 2e). This suggests that in addition to the feed additives, the basal medium can substantially contribute to the ammonium metabolism of the cells. Despite lower cell concentrations in OptiCHO, ammonium increased drastically during the stationary phase of the cultures (Fig. 2e) with occasionally sixfold higher specific production rates than in ActiCHO P (data not shown). Recently, the production of ammonium and also alanine was linked to the consumption of aspartate and glutamic acid in a glutamine synthetase CHO cell line (Duarte et al. 2014). In our experiments, similar observations were made for the concentration of alanine, which increased in all cultures. Furthermore, higher Asp consumption was found in OptiCHO fed-batches than in ActiCHO P cultures, which makes alanine a potential source for ammonium. However, in our case, the specific Glu consumption rates did not correlate well with the specific ammonium production rates (Table 4).

Although low lactate concentrations are generally desired in bioprocessing, our data and work previously published by Li et al. (2012) showed that a total depletion can coincide with increased ammonium levels. Xing et al. (2008) found that ammonium levels above 5.1 mM (92 mg/L) can inhibit cell growth. This threshold was passed on day 7 in OptiCHO fed-batches and indeed coincided with peak cell concentrations that declined thereafter. However, cell viability and antibody production were not negatively impacted.

Cell cultures grown in OptiCHO and supplemented with Feed A and B accumulated glutamic acid, ammonium, and lactate throughout the process and the concentrations were higher than in any other fed-batch (Fig. 2d–f). In parallel, the osmolality increased steadily (Fig. 2c). In most cell culture media, the osmolality ranges between 270 and 330 mOsm/kg, and it has been shown that increased levels can severely reduce specific cell growth rates (Kim et al. 1998; Kim and Lee 2002; Zhu et al. 2005). The suggested inhibitory threshold for affecting cell viability negatively was reported to be 380 mOsm/kg (Xing et al. 2008). In two of the evaluated fed-batch cultures this osmolality was exceeded 1 day after the end of the exponential growth phase, and this observation coincided with declining cell concentrations. However, the cell viability was not affected and remained above 90 % for the following 4 to 6 days (data not shown). The steadily increasing osmolality in OptiCHO fed-batches supplemented with Feed A and B was most likely the result of a constant feeding regimen. Already on day 5, 15 of 18 measured amino acids were 200–500 % higher than in the original medium composition and the remaining three close to their original concentrations (Table 3C). As discussed above, this might have induced cellular stress, leading to a less efficient glucose metabolism and the resulting high lactate concentration (Fig. 2d). This hypothesis is supported by the twofold to fourfold increase in cell-specific lactate production compared with the other fed-batch strategies studied, despite similar specific glucose consumption rates.

Protein glycosylation is an important post-translational modification, as sugar residues are involved in many processes such as recognition and biological regulation. The glycan structures also determine certain properties of the recombinant protein including immunogenicity, efficacy, and serum half-life. Therefore, it is desired to control glycosylation tightly, thus contributing to constant quality of the recombinant glyco-protein. Cellular glycosylation can be affected by numerous factors like the host cell line, protein structure, medium components, and culture conditions (Butler and Spearman 2014). The three main glycan structures observed in our experiments were G0F, G1F, and G2F (Fig. 3). Similar observations were reported previously (Costa et al. 2013). Core fucosylation is a typical attribute of CHO-expressed proteins. However, studies have shown that defucosylated antibodies exert an up to 100-fold improved antibody-dependent cellular cytotoxicity (ADCC) (Shields et al. 2002) and defucosylated glycans are therefore desired for therapeutics that rely on this effect. Increased amounts of high mannose structures may reduce ADCC and complement-dependent cytotoxicity (CDC) (Kanda et al. 2007). Additionally, high mannose structures can result in higher clearance rates (Goetze et al. 2011). The levels of high mannose glycans for endogenous human IgG is less than 0.1 % but can range from 1 % to greater than 20 % in recombinant antibodies (Flynn et al. 2010). Low to medium levels of mannose 5 (Man5) modified IgG (3 to 9 %) were observed in our experiments. In general, the concentration of high mannose structures was reduced by about 50 % in cultures grown in ActiCHO P (Fig. 3). Terminal galactosylation is not yet fully understood (Costa et al. 2013). However, as agalactosylated (G0) structures in antibodies have been associated with several pathologies (Parekh et al. 1985), higher galactosylated (G1, G2) antibodies are generally preferred. The glycan distribution with the lowest G0F, but highest G1F/G2F structures, were found in ActiCHO P supplemented with Feed A and B, and in OptiCHO fed with EfficientFeed A for 9 days (Fig. 3). However, the higher Man5 percentage found in antibodies in the latter combination should be considered critical in case of in vivo experiments.
